# Bumblebees avoid sucrose solution containing high concentrations of Roundup

**DOI:** 10.1007/s10646-025-02878-9

**Published:** 2025-03-31

**Authors:** Linzi Jay Thompson, Dara A. Stanley, Marie Dacke, Lina Herbertsson

**Affiliations:** 1 School of Agriculture and Food Science, University College Dublin, Dublin, Ireland; 2 Earth Institute, University College Dublin, Dublin, Ireland; 3 Vision Group, Department of Biology, Lund University, Lund, Sweden

**Keywords:** Bees, Glyphosate, Pesticide, Ecotoxicology, Weedkiller

## Abstract

Herbicides are one of the most heavily applied groups of pesticides globally. Whilst research on herbicides in relation to bees is growing, we still have more to learn about how bees may interact with herbicides and the subsequent consequences for bee health. As herbicides are designed to kill the plants they are applied to, bees and other pollinators may interact with them in a different way to other pesticide groups which is important to understand in the context of evaluating hazard and risk. Here, we conducted both a choice and no-choice test, to determine if bumblebees would be deterred from foraging from feeders containing commercial formulations of Roundup (Ultra and Biactive, respectively) compared to controls. We found across both experiments that bees were deterred from foraging where feeders contained above field-realistic concentrations of Roundup formulation, and that on average colonies reduced their consumption from these feeders by ~50% despite lacking other food sources. This demonstrates that, when given no choice, bees can be deterred from sucrose containing Roundup Biactive, although above expected field concentrations, even to their own nutritional detriment. Separately, individual foragers were observed avoiding feeders containing field-realistic levels of Roundup Ultra compared to controls, showing a preference for uncontaminated feed when given a choice. As this was an experimental setup using high concentrations of Roundup with sucrose solution rather than real flowers, more work is needed to understand this phenomenon under field conditions. This work provides useful information and insights for future studies investigating the impacts of glyphosate in the form of both active substance and formulation on bees and could also be useful in identifying future mitigation strategies for field use.

## Introduction

Bees can be exposed to a variety of pesticides whilst foraging for nectar and pollen. Herbicides, aka ‘weed killers’, are a group of pesticides designed to target weeds (FAOSTAT, 2020). Glyphosate-based herbicides, including Roundup, are among the world’s most commonly used herbicidal formulations and act by inhibiting the 5-enolpyruvoylshikimate 3-phosphatesynthase enzyme, which is only present in plants (Baer and Marcel 2014). Yet, there is evidence that Roundup and other formulations with glyphosate also affect bees (e.g. Abraham et al. 2018, Battisti et al. 2023, Straw et al. 2021). Observed adverse effects of Roundup and glyphosate include multiple physiological functions, and the number of studies on this topic has increased during previous years (Battisti et al. 2023). Studies where bees have been exposed to Roundup or glyphosate in the field are rare, but some evidence suggests that field exposure to Roundup can delay brood development (Odemer et al. 2020), highlighting the need to evaluate the impact of field-realistic concentrations of Roundup and glyphosate. However, most of the studies have been performed on *Apis mellifera* (Battisti et al. 2023), highlighting a need to expand the research beyond this single species. Bees can be exposed to residues whilst foraging (Thompson et al. 2014; 2022) because glyphosate remains present in nectar and pollen of treated plants that continue to attract pollinators for several days after glyphosate treatment (Thompson et al. 2014; 2022), thereby putting bees at risk of repeated exposure. However, recent studies indicate that pollinators visit plants treated with low concentrations of glyphosate (active substance) less often than untreated plants (Russo et al. 2020) and that glyphosate (active substance), while not entirely preventing bees from foraging, reduces the amount of time bees spend collecting nectar (Thompson et al. 2022). Together this suggests that low levels of glyphosate may have some deterrent effect, potentially mitigating harmful effects of high exposure.

Herbicidal treatment of plants typically involves applying glyphosate as part of commercial formulations, and not solely active substances. Co-formulants can have impacts on bees unrelated to the active substance (e.g. Cullen, et al. 2023; Straw et al. 2022), yet we have very little knowledge of which co-formulants are commonly used and how these behave in the environment. For a better understanding of the effect of glyphosate-based formulations on pollinators we need to address how pollinators respond to these formulations, as well as to the active substances in isolation. In an ideal scenario, we would evaluate the effects of individual co-formulants; however, this information is generally unavailable for formulations and as such the next best approach is to study the formulation in its entirety. When considering the impact of pesticides on pollinators it is important to evaluate more than the lethal and sub-lethal impacts, but also to understand how bees may become exposed. In this study, we assessed if foraging bumblebee individuals (*Bombus terrestris L*.) are repelled by sucrose solution with the glyphosate-based herbicidal formulation Roundup Ultra, and whether bumblebee colonies provided with sucrose containing Roundup Biactive consumed less than those fed control sucrose, using choice and no-choice tests respectively to evaluate bumblebee preferences.

## Materials and methods

### Choice test

One colony of *B. terrestris*. (Koppert) was placed in an indoor cage, 2.1 × 2.6 × 1.7 m, in a behavioral lab for insect studies at Lund University, Sweden. The temperature was set to 22 °C and humidity was around 36%. Lights were automatically set to be on between 7 am and 6 pm. Bumblebees were fed sucrose solution from two identical feeders consisting of a 100 mL plastic tube, with sucrose solution (40% w/w), accessible via a piece of cotton through the yellow lid of the plastic tube (Fig. S1). During the training phase, the feeders were initially placed just outside the hive until the bees had learned to find them and we slowly increased this distance up to around 1.5 m from the hive (underneath a camera mounted on the top of the cage), where we put them during the experiment. We tagged the bees with numbered honeybee queen-marking tags, to distinguish individuals. We were not able to tag all individuals visiting the feeders, as new workers were constantly recruited. During data collection, one of the feeders contained Roundup Ultra (Monsanto, isopropylamine salt of glyphosate, CAS number 38641-94-0, 360 g glyphosate acid/L, batch code AXD260911, production date 27 April 2017), while a control feeder contained unspiked control sucrose solution. Roundup Ultra was registered in Sweden at the time of the experiment (The Swedish Chemicals Agency 2024). The concentration of Roundup varied among experimental rounds and corresponded to 0.04–125.93 mg a.s./L which is in the field-realistic range, i.e. what has been measured in pollen and nectar from treated flowers (Thompson et al. 2014; 2022, Zioga et al. 2022). At the highest concentrations (>60 mg a.s./L), we quickly noticed that the bees preferred the control sucrose, but because we were interested in understanding if they were repelled by Roundup also at lower concentrations and where a potential cutoff point occurred, we prioritized shorter distances between tested concentrations at the lower end of the range. Our initial plan was to run similar number of trials per concentration, but because the outbreak of COVID-19 interrupted the study, we performed four trials per selected concentration for 0.04, 4.20, 8.40, 12.60 and 16.80 mg a.s./L, two trials per concentration for 2.10, 52.47, 104.94 and 125.93 mg a.s./L and six trials for 20.99 mg a.s./L. All trials were run in pairs with shifted position of the Roundup and control feeders, to avoid that a potential preference for a certain feeder position influenced the results. We weighed the feeders before placing them in the cage, next to each other, at equal distance from the nest. Simultaneously, one feeder was prepared, weighed and placed next to the cage to account for evaporation. The bumblebees had access to the feeders for 60 min, starting when the first bee landed on a feeder. When the time had passed, we closed the hive, so that the bees could only enter, not exit. We waited until all bumblebees had left the feeders before the feeders were removed. This was done to avoid accidental reduction in sucrose solution when manually removing the bees. We weighed the feeders and washed them with hot water, detergent and alcohol. We subtracted weight loss in each feeder with the evaporation for each of the two feeders. After this, we calculated the proportional consumption from the Roundup feeder compared to the control feeder (expecting 0.5 from each if bees select at random) and evaluated its effect on feeding by specifying a beta regression model (R package “betareg”, Grün et al. 2012) using R version 4.4.1 (R Core Team 2024).

During 27 out of 34 trials, we video recorded the feeders from above, to track the foraging of individual bumblebees. We used the video recordings to track the visits by individually tagged bees to the feeders (Fig. S1). We counted any visit where the bee was feeding, and noted the time it started feeding which generally occurred immediately. While it was usually easy to detect the start of the feeding event, it was often difficult or impossible to determine when the bees stopped feeding. Instead, we noted when they left the feeder, more specifically the yellow lid, or the cotton (Fig. S1). To understand if the bees learned to avoid the Roundup feeder during the trial, we tested if the probability of bees to choose the Roundup feeder depended on the concentration of Roundup and the time since the trial started. We did this with a generalized linear mixed model (R package “lme4”, Bates et al. 2015), with feeder (Roundup or control) as the dependent variable, (z transformed) concentration of Roundup (mg a.s./L) and (z transformed) landing time, as well as an interaction between these as independent variables, and trial and bumblebee ID as crossed random factors. Landing time and concentration were z transformed to allow model convergence.

To test if bees spent less time on feeders that contained Roundup Ultra and if this difference increased with increasing concentration of Roundup (a.s. mg/L), we fitted a linear mixed model with the (square root transformed) length of these visits as response variable. The length of the visits was averaged per bumblebee ID and trial and thereafter square root transformed to improve the residuals. We added feeder (Roundup Ultra or control), concentration of Roundup Ultra (mg a.s./L) and the interaction between these two as explanatory variables, and trial as random factor. For the mixed models, we estimated p values using Wald’s chi-square test (R package “car”, Fox and Weisberg, 2019) and kept the two main term effects and their interaction in the model irrespectively of their estimated effect.

We ran all models with and without the three highest concentrations, because of the low sampling effort at this end of the gradient (two trials each for all three concentrations). All results from analyses using the full, as well as reduced, dataset are presented in the results section and figures. For the smaller subset of the data, all concentrations were within the field-realistic range of what has been measured in nectar (Thompson et al. 2014). For consumption, the residuals from this betareg model were sub-optimally distributed and we therefore verified the results with a linear model from the package “stats” (R Core Team 2024), where we arcsin square root transformed the response variable, due to its proportional nature. For landing probability, bumblebee ID did not contribute with any variance to the model with the reduced dataset and was removed from the random structure. We evaluated residuals from all models visually and extracted confidence intervals with the package “effects” (Fox and Weisberg, 2019).

### No-choice

The no-choice experiment was performed at University College Dublin, Ireland. Colonies (*n* = 24 total/ 6 per treatment, Biobest, Westerlo, Belgium) of *B. terrestris* received ad lib sucrose solution (40% w/v) spiked with either 1 mg a.s./L glyphosate (>95% purity, CAS number 1071-83-6; Molekula), low concentration of Roundup Biactive XLA (Monstanto, potassium salt of glyphosate, CAS number: 70901-12-1, 360 g glyphosate acid/L, batch code AZL100810A, production date 10/11/2018), corresponding to 1 mg glyphosate/L (1 mg a.s./L)) or high concentration of Roundup Biactive XLA (21,600 mg a.s. /L, batch details as above), or unspiked sucrose solution as a control. Feeders were accessed by bees within the colony box, in a similar way to their commercial nectar substitutes. The colony box was removed from the cardboard casing and covered in paper to provide bees with darkness (Fig. S2). Each colony was given 2 days of access to a foraging arena, where they could fly freely. Each arena contained a feeder of their respective treatment solution (made of petri dishes with mesh over the top) (Fig. S3). The selected concentrations were chosen to mimic a range of exposure scenarios. The 1 mg a.s./L concentration was chosen as an environmentally relevant concentration which bees may be exposed to when foraging from treated plants (Thompson et al. 2014, Thompson et al. 2022), while we also included the maximum spray rate concentration of the commercial formulation (21,600 mg a.s./L). It is unlikely that bees would be exposed to this spray rate concentration as residue in nectar and pollen; however, as this is a concentration which is applied to plants (including those in flower) it is possible that this could be a worst-case scenario, particularly where large areas of land are treated. Colonies were weighed before pesticide exposure and distributed across treatments to ensure an even balance of colony weight across treatments, these were then structured into cohorts of four containing one of each pesticide treatment balanced by their weight. Feeders were weighed and refilled every 1–2 days over a five-day period. One colony was removed from the Roundup high treatment due to consistent leakage of the feeder (*n* = 5); removal of this data point did not change the model conclusions.

We analyzed if the total and daily consumption, respectively, differed among treatments using linear mixed effects models, from the lme4 package (Bates et al. 2015) with R version 4.4.1 (R Core Team, 2024). Daily consumption was square root transformed to obtain optimal residuals. For daily consumption we added day (integer) and an interaction between day and treatment to test if the effect of the treatment changed over time. We specified cohort as random factor in both models and for daily consumption, where we had five values per colony, we nested colony within cohort. We evaluated residuals visually. The significance of factors and the interaction was checked with Type II Wald chi-square test (R package “car”, Fox and Wei, 2019) and we evaluated differences among factor levels using a tukey test from the emmeans function from the emmeans package (Lenth, 2024).

## Results

### Choice test

**Fig. 1:**
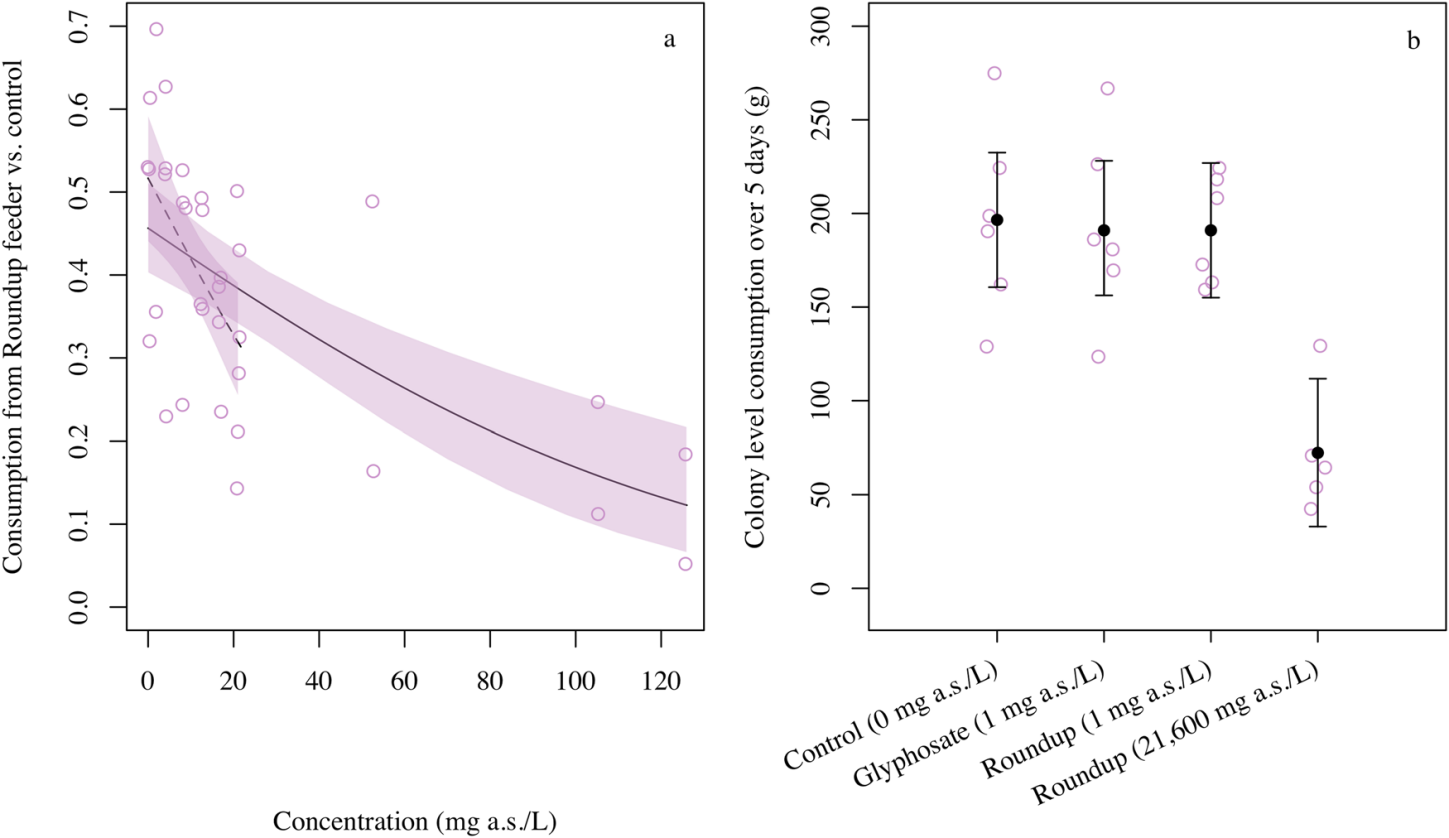
Consumption of sucrose solution with different concentration of glyphosate or Roundup, specified as mg a.s./L sucrose solution. **a**) proportional consumption from feeder with spiked sucrose solution (Roundup Ultra) in the choice test. The plot shows model estimated means for the full dataset (solid line) and without the three highest concentrations (black dashed line), 95% confidence intervals (shaded areas) and raw data (open circles), **b**) consumption of sucrose solutions by bumblebee colonies over a five-day period from the no-choice test (Roundup Biactive). Model estimated mean (filled circles), 95% confidence intervals (whiskers) and raw data (open circles) are plotted for each treatment.

At the trial level, we observed that the consumption of sucrose solution from the feeder with Roundup Ultra compared to the control feeder declined with increasing concentration of Roundup *(pseudo-R2* = *0.468*, *z* = *−4.504*, *p* < *0.001*, Fig. 1a) and this was unaffected by the removal of the three highest concentrations (*betareg: pseudo-R*^*2*^ = *0.274*, *z* = *−3.215*, *p* = *0.001, pseudo-R2* = *0.274*, Fig. 1a*, LM: R*^*2*^ = *0.277*, *t* = *−3.157*, *p* = *0.004*). The 95% confidence interval was overlapping with 0.5 at concentrations below 2.44 mg of a.s./L for the full dataset, and below 7.09 mg of a.s./L, when excluding the three highest concentrations (Fig. 1a). We observed that increasing concentration of Roundup Ultra, in combination with increasing time since the start of the trial, reduced the probability of tagged bumblebees to land on the Roundup feeder (concentration ⋅ time: χ^2^_*1*_ = *3.856*, *p* < *0.050*, Fig. S4). When excluding the three highest concentrations, this interaction was not significant (concentration × time: *χ*^*2*^_*1*_ = *0.464*, *p* = *0.496*) and time since the start of the trial also had no effect (*χ*^*2*^_*1*_ = *2.403*, *p* = *0.121*), whereas the probability that a bee landed on the feeder with Roundup Ultra declined marginally with increasing concentration (*χ*^*2*^_*1*_ = *3.222*, *p* = *0.073*). Visits to the Roundup feeder were shorter than to the control feeder and this effect increased with increasing concentration of Roundup Ultra (feeder × concentration: χ^2^_*1*_ = *12.795*, *p* < *0.001*, Fig. S5), also when excluding the three highest concentrations (feeder × concentration: *χ*^*2*^_*1*_ = *7.438*, *p* = *0.006*, Fig. S5). Random effects for these models were positive.

### No-choice test

In the no-choice experiment, both total consumption (*χ*^*2*^_*3*_ = 32.947, *p* < 0.001, Fig. 1b) and daily (*χ*^*2*^_*3*_ = 49.911, *p* < 0.001, Fig. S6) consumption differed among treatments. Day had no influence on daily consumption, neither alone (*χ*^*2*^_*1*_ = 0.67, *p* = 0.410) nor in interaction with treatment (*χ*^*2*^_*3*_ = 0.580, *p* = 0.901). For total as well as daily consumption, the difference among treatments was entirely driven by lower consumption of the Roundup high contaminated solution, which differed significantly from all other treatments (Tukey: *p* < 0.002, Table S1, 2), and none of the other treatments differed from each other (Tukey: *p* > 0.99, Table S1, 2).

## Discussion and conclusions

We show that individual bumblebees avoid feeding on sucrose solutions containing Roundup Ultra (active substance: glyphosate, concentration ranging from field-realistic to higher) when they have a choice between Roundup spiked and control sucrose solution. The bees spent less time on the Roundup feeder and that the length of these visits declined with increasing concentration of Roundup, showing a repellent effect of Roundup. This result was robust to the removal of the three highest concentrations. When excluding concentrations above 25 mg a.s./L, we observed that the bees landed marginally less often on the feeder containing Roundup Ultra compared to the control feeder. This effect was significant when including higher concentrations and the inclusion of these also revealed that the bees developed an increasing preference to land on the control feeder within the 60 min of each trial. This pattern was however, only observed when concentrations above field-realistic were included. Because the experiment was performed on group-feeding bumblebees, we cannot tell if this process reflects the learning by individual bees or the group, as bumblebee workers are known to learn behaviors from each other (Bridges et al. 2024). As bees spent more time on the control feeder, this may have resulted in higher bee densities, attracting an increasing number of bees to this feeder throughout the trial. Independently of the mechanisms, the fact that the bees - at high concentrations of Roundup Ultra - developed a preference to land on the control feeder brings up an interesting and potentially important question about how this translates into field conditions, where – on one hand – the position of treated versus untreated plants is less variable, allowing bees to learn where to forage, whereas – on the other hand – the distances between them may be longer, reducing the available alternatives. It is, however, important to note that the concentrations where we detected a time-dependent effect were above field-realistic.

The repellent effect of Roundup Ultra was greater with increasing concentration, possibly mitigating exposure to high concentrations of Roundup Ultra in the field, as bees may avoid foraging on plants contaminated with higher concentrations. Due to the complexity of real environments, with floral scents masking or diluting the odor or taste of the herbicide, thus potentially reducing the bees’ ability to detect and avoid Roundup, this result needs to be verified in the field. In our study, the model predicted significantly reduced consumption from the Roundup feeder at any concentration above 2.44 mg of a.s./L, which is within the field-realistic span (Thompson et al. 2014). As a comparison, honeybees that were only given access to glyphosate treated *Phacelia*, brought back nectar with higher concentrations than this during the first week after spraying (Thompson et al. 2014). Another study, where honeybee colonies were placed in the field adjacent to 1 ha of treated Phacelia reported much lower concentrations, up to 0.5 ± 0.5 mg/kg in food storages in the hives, despite that concentrations in the treated plants were around 100 times higher (Odemer et al. 2020). If our result reflects a true behavior among bees under field conditions, it could be anticipated that an ability to avoid Roundup Ultra reduces the risk of exposure to high concentrations that may occur after spraying (Thompson et al. 2014; 2022), potentially explaining the relatively low exposure when bees have access to alternative forage areas (Odemer et al. 2020). Because higher concentrations of Roundup and a.s. glyphosate can have adverse effects on bee behavior, for example by reducing the ability to discriminate among colours in bumblebees (Helander et al. 2023) and homing success in honeybees (Balbuena et al. 2015), an ability to detect and avoid concentrations above 2.44 mg of a.s./L may be beneficial for foraging bumblebees. However, even lower concentrations, from which the bees were not repelled in our study, can affect bumblebees, for example by changing the gut microbiota in bumblebees (e.g. Cullen et al. 2023) and delaying brood development in honeybees (Odemer et al. 2020). In honeybee colonies, nurse bees were recently shown to play a critical role in reducing the broods’ exposure to fungicide residues (Wueppenhorst et al. 2024), and it is possible that avoidance of Roundup in a similar manner protects the developing brood from harmful concentrations, potentially mitigating negative effects on colony health and populations, but this needs to be further studied. It is important to underline that we have only tested two Roundup formulations and that it is currently unclear how representative they are for other formulations. More importantly, although there is plenty of evidence for sublethal effects of Roundup and glyphosate, of which some can be expected to reduce bee fitness and survival (Battisti et al. 2023), we have not found any clear evidence supporting a direct link between exposure to Roundup under field conditions and adverse effects on bee health and populations. Because of our approach, where the same bees were presented the two feeders (Roundup Ultra or control) at multiple occasions and we alternated the position of the Roundup feeder between each trial, the bees were likely to start each trial with a preference for the Roundup feeder and we may thus have underestimated the bees’ unwillingness to consume sucrose solution containing Roundup Ultra. It is also possible that repeated exposure to high levels of Roundup Ultra influenced the bees’ response, either by increasing or reducing their willingness to consume this sucrose, which we cannot conclude from this experiment.

Interestingly, we observed a similar, dose-dependent, effect of Roundup Biactive (active substance: glyphosate) at the colony level. Whilst the field-realistic concentration of both Roundup Biactive and glyphosate active substance had no impact on consumption, the colonies exposed to the higher concentration of Roundup Biactive responded by reducing their consumption by ~50%, even though they had no other food source available. We cannot exclude that this effect was driven by reduced appetite, potentially via decreased activity and energy demands and whilst there is some evidence that food consumption by honeybees is unaffected by chronic exposure to technical grade glyphosate (Herbert et al. 2014), it is - to our knowledge - unclear whether exposure to Roundup formulations can affect bee appetite. However, if the observed effect on sucrose consumption were to be driven by reduced appetite, we would expect an increasing difference among treatments over time. Because the reduction was constant, we suggest that the lower consumption resulted from bees being deterred from consuming sucrose with high concentrations of both Roundup formulations. While reduced consumption of sucrose solution with high concentrations of Roundup Ultra or Biactive of course mitigates exposure, a strong reduction in food resources being returned to the colony could have a detrimental effect on colony health, particularly in relation to their reproduction (Osbourne and Goulson, 2017). Such high concentrations may, however, be unlikely for bees to consume orally in the field as the herbicide is likely to be absorbed rapidly into the plant or become diluted in nectar. For example, honeybees foraging from glyphosate treated *Phacelia* collected nectar with up to around 30 mg a.s./kg (note the slightly different unit compared to our study, Thompson et al. 2014), which is less than a percentage of the highest concentration in our no-choice experiment. In this context, it is also relevant to acknowledge that while there is a risk that avoidance of extreme concentrations of Roundup reduces foraging efficiency, the ultimate reason for applying glyphosate-based formulations is to control weeds, which can be expected to reduce the availability of floral resources within a few days (e.g. Thompson et al. 2022) and consequently make foraging less efficient.

Why these concentrations of two different Roundup formulations result in bees choosing to avoid food resources is also unclear: whether this is driven by changes to physical properties, or deterrence caused by a shared co-formulant, or glyphosate itself, remains unknown. Interestingly, (Thompson, et al. 2022) observed similar flower-visitation frequency to untreated flowers and flowers sprayed with 3400 mg a.s. /L, but nectar foraging events were noticeably shorter to the treated plants, suggesting a slightly repellent effect of glyphosate, which was not strong enough to entirely prevent the bees from foraging. Liao et al. (2017) found no significant repelling effect on honeybees of sucrose solution with glyphosate 10 mg a.s./L, but found a significant preference towards glyphosate treated sucrose solution at 0.01 mg a.s./L. This could suggest that the driver behind our observed deterrence could be a result of co-formulants or potentially differences in the preferences of bee species. It is possible that the deterrence is a result of changes to the physical properties rather than simply the presence of a co-formulant or active substance. During the no-choice test, at the highest concentration (21,600 mg a.s./L) of Roundup Biactive, we noticed changes in the properties of the solution, including a darkening in colour (Fig. S3), a change in scent (stronger odour of the Roundup Biactive solution, anecdotally) and reduced surface tension or viscosity. For this reason, all feeders from the high concentration required a plate underneath to capture any drops that leaked, slowly, but continuously, which was not needed in any other treatment. It is unclear to what extent this affected the bees’ ability to feed and therefore the deterrence could, at least in part, be a result of a difficulty consuming the solution. Changes in properties were observed at the highest concentration but not quantified, and as such we cannot determine to what extent they occurred in the lower concentrations. As it is unknown which co-formulants are present in both Roundup Ultra and Biactive, we cannot determine the likelihood of co-formulants migrating into floral resources following spray application. Although it would be beneficial to determine how the physical properties of nectar change following application of Roundup formulations, this would likely be challenging due to the small volumes of nectar in individual flowers.

The bumblebees’ unwillingness to consume sucrose with Roundup Ultra at field-realistic levels, and Roundup Biactive at higher concentrations is of particular importance for researchers who are assessing effects of pesticides on bees, where unawareness can result in lower intake of sucrose and pesticides than expected. Starving effects caused by avoidance of Roundup Biactive-spiked sucrose solution can potentially also be misinterpreted as detrimental effects of exposure to the formulation. For example, a general observation was made that when given access to foraging arenas, bees from the above field-realistic concentration of Roundup Biactive solution were lethargic and were often observed still, next to feeders with very low activity. While this was observed for all colonies at the highest concentration, it was not observed in any of the field-realistic concentrations (Fig. S4). Due to the extremely reduced sucrose consumption, we cannot tell if this was an effect of starvation or of exposure to the formulation. This research highlights that bees may choose to starve or utilize their colony resources rather than forage, demonstrating that measuring consumption is a key part of any pesticide research to help pick apart these effects.

The results from the choice test show that where low concentrations of Roundup Ultra are present in sucrose solution, it is possible that - whilst bees may be deterred from foraging - resource collection could still occur, and that when given no-choice, bumblebees will consume equal amounts of control sucrose and sucrose spiked with Roundup Biactive, at field realistic concentrations. Subsequently we need more research to better understand the environmental fate and effects of pesticide formulations on bee health at individual, community and population levels, especially at field-realistic concentrations. While our study contributes with some evidence that bees can avoid field-realistic concentrations of Roundup Ultra when they have a choice, and even higher concentrations of Biactive when having no choice, there is a need to understand how other comparable products influence foraging preferences, not least in the field, where the odor or taste from the product may be covered by floral scents, and distances between treated and untreated flowers are longer. It is also important to understand if the ability and willingness to avoid high concentrations of Roundup Ultra and Biactive are unique to commercially available *B. terrestris*, or if wild *B. terrestris* and other bumblebee species respond similarly.

The observed reduction in consumption of Roundup formulations could on one hand reduce the risk of exposure, providing some mitigation from the effects of glyphosate and co-formulants, but might on the other, cause nutritional stress where alternative food sources are unavailable, e.g. where Roundup is used to clear large areas of land. However, is it also important to acknowledge that the loss of floral resources following such treatment may have a stronger influence on nutritional stress. To better understand our results in the context of risk assessments and potential mitigation strategies we need to further our understanding of how the avoidance of Roundup-spiked sucrose solution translates into field realistic scenarios and explore whether this phenomenon exists in other formulations.

## Supplementary information

Supplementary material

## Data Availability

Data associated with this manuscript can be found on Figshare at: 10.6084/m9.figshare.21904623.
