# Supplementary material for: Bumblebees avoid sucrose solution containing high concentrations of Roundup
Source: Ecotoxicology. 2025 Mar 31;34(5):845–52. doi: 10.1007/s10646-025-02878-9 (PMC12254069; doi:10.1007/s10646-025-02878-9)
Supplement: Supplementary file 1 — Supplementary material [file 10646_2025_2878_MOESM1_ESM.pdf]

Supplementary material (S1-S6) for

Bumblebees avoid sucrose solution containing high concentrations of Roundup

Linzi Jay Thompson [1,2], Dara A. Stanley[1,2], Marie Dacke[3] and Lina Herbertsson[3]

1. School of Agriculture and Food Science, University College Dublin, Ireland

2. Earth Institute, University College Dublin, Ireland

3. Vision Group, Department of Biology, Lund University, Sweden

Table S1 Pairwise comparisons for total consumption, significant differences ($P < 0.05$) highlighted in bold.

Contrast	Estimate	SE	df	Lower CI	Upper CI	t-ratio	p-value
Control – Glyphosate AI	4.38	24.0	14.1	-65.5	74.2	0.182	0.9977
Control – Roundup high	124.20	25.5	14.7	50.6	197.8	4.873	**0.0011**
Control – Roundup low	5.58	24.0	14.1	-64.3	75.4	0.232	0.9954
Glyphosate AI – Roundup high	119.82	25.5	14.7	46.2	193.5	4.701	**0.0015**
Glyphosate AI – Roundup low	1.20	24.0	14.1	-68.7	71.1	0.050	1.0000
Roundup high – Roundup low	-118.62	25.5	14.7	-192.3	-45.0	-4.654	**0.0017**

Table S2 Pairwise comparisons for daily consumption, significant differences ($P < 0.05$) highlighted in bold.

Contrast	Estimate	SE	df	Lower CI	Upper CI	t-ratio	p-value
Glyphosate AI - Roundup high	2.5550	0.443	14.3	1.27	3.84	5.765	**0.0002**
Glyphosate AI – Roundup low	-0.0592	0.416	13.9	-1.27	1.15	-0.142	0.9989
Glyphosate AI – Control	-0.0981	0.416	13.9	-1.31	1.11	0.236	0.9951
Roundup high – Roundup low	-2.6142	0.443	14.3	-3.90	-1.33	-5.898	**0.0002**
Roundup high – Control	-2.6531	0.443	14.3	-3.94	-1.37	-5.986	**0.0002**
Roundup low - Control	-0.0389	0.416	13.9	-1.25	1.17	-0.094	0.9997

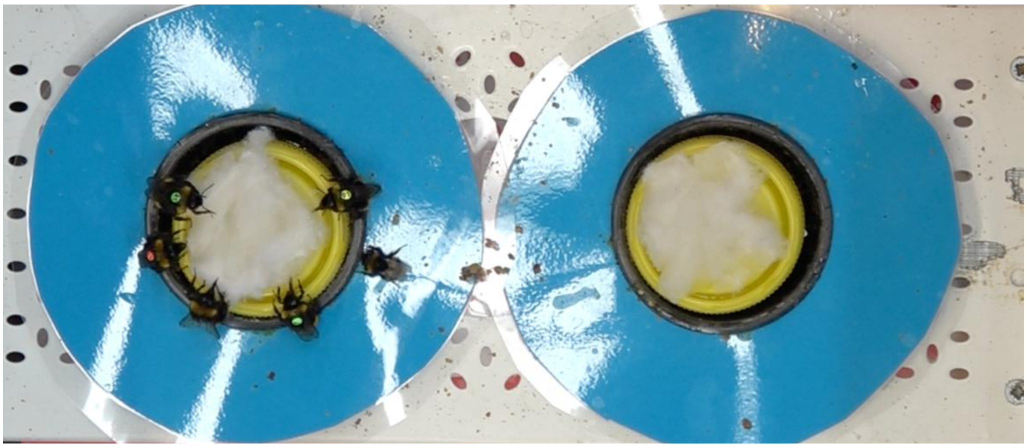

Figure S1 The feeders that were used in the choice experiment. The feeders were always placed at the same distance from each other, but the position of the Roundup Ultra spiked versus control feeder was alternated between each trial. All components of the feeder were thoroughly washed with hot water, detergent and alcohol between trials.

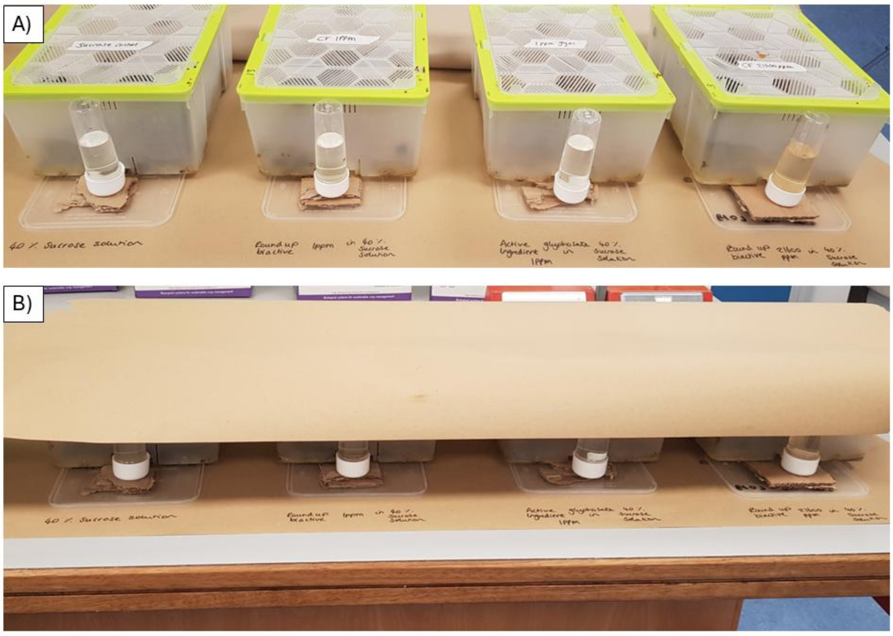

Figure S2 The set up of the no-choice colony experiment. In A) the feeders for each treatment can be seen, from left ro right: Sucrose control, Roundup Biactive 1 ppm, Active ingredient glyphosate 1 ppm and Roundup Biactive 21,600 ppm - note the change in colour. B) Shows how colonies were typically reared - covered in a sheet to keep them dark, bees were only exposed to the light during initial set up.

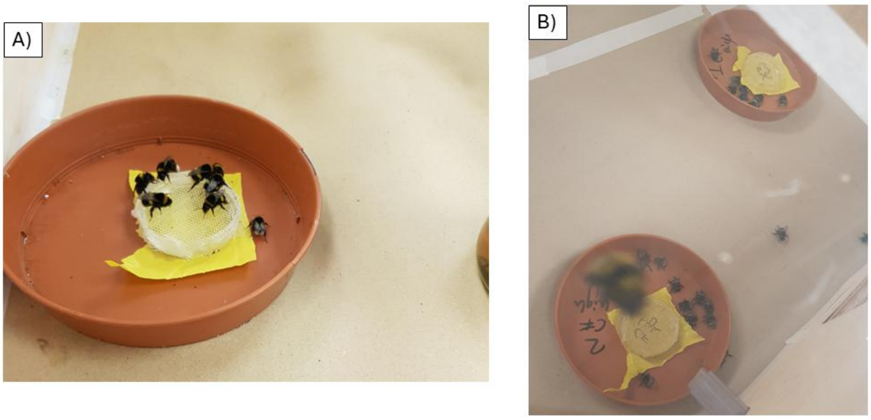

Figure S3 Bees foraging in flight arenas on their treatment solutions. A) shows bees from the control solution actively foraging. B) shows bees from the Roundup Biactive high treatment, sitting next to feeders and not consuming - bees with

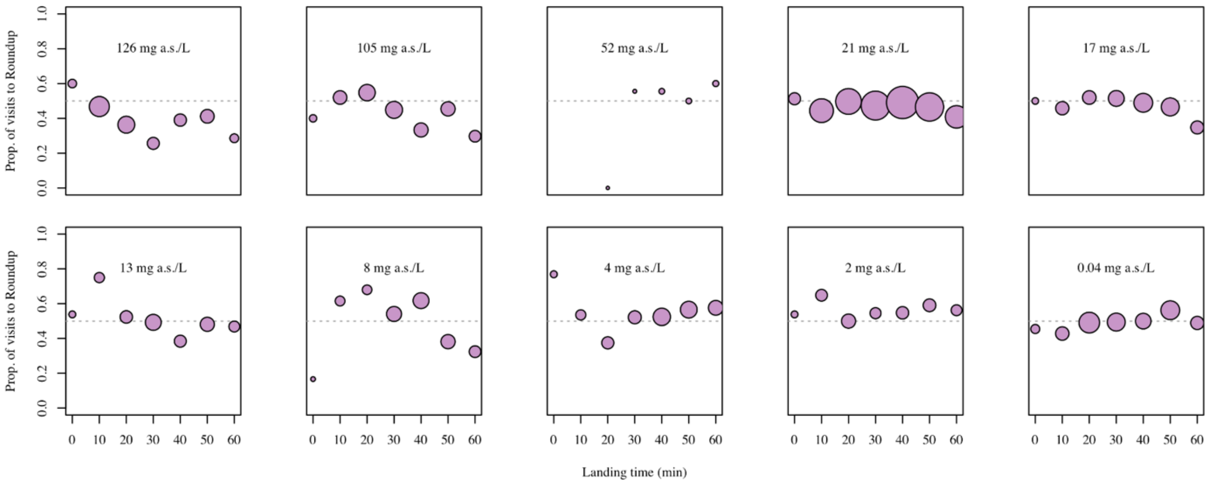

Figure S4. The probability of tagged bumblebees to land on the feeder with Roundup Ultra, compared to the control feeder (no Roundup), was affected by an interaction between concentration and time since start of the trial (concentration · time: $\chi^2_1 = 3.856, p < 0.050$). More specifically, for the higher concentrations, but not the lower concentrations, the proportion of landings on the Roundup feeder declined over time, and when excluding concentrations at 52 mg a.s./L and higher, the interaction between concentration and time was no longer significant ($\chi^2_1 = 0.46, p < 0.496$). For this subset of the data, the probability to land on the feeder with Roundup Ultra decreased marginally ($\chi^2_1 = 3.22, p < 0.073$) with increasing concentration of Roundup Ultra, whereas landing time had no influence on the probability to land on the feeder with Roundup Ultra ($\chi^2_1 = 2.40, p = 0.121$). The circles, which are proportional to the total number of visits to the two feeders (Roundup and

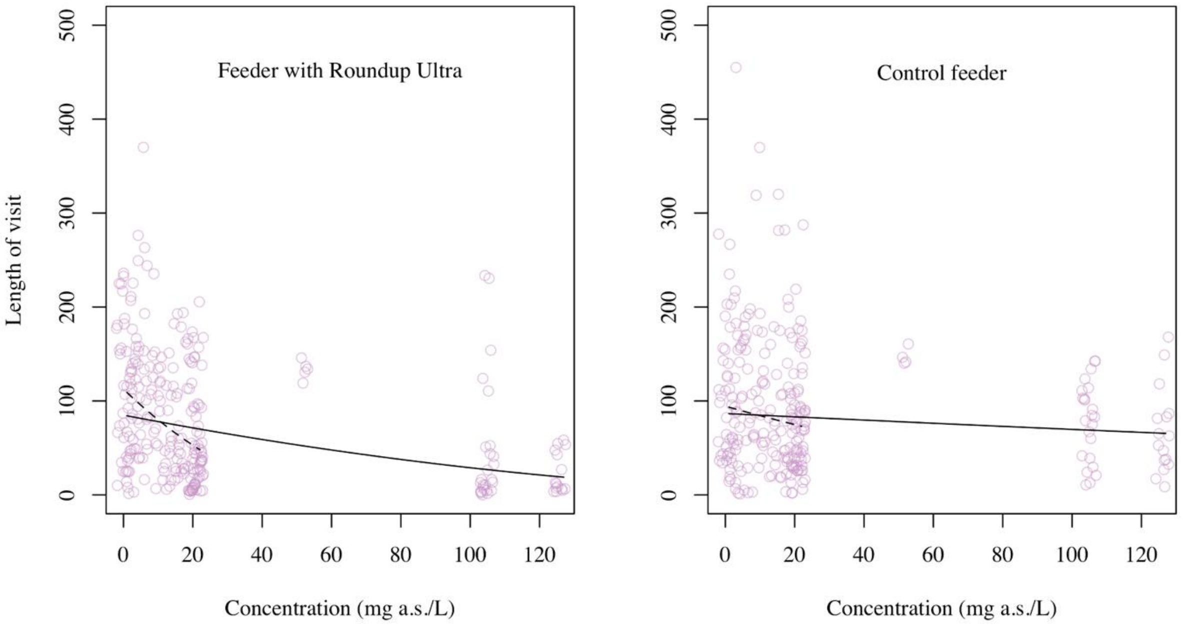

Figure S5 When tagged bees landed on the feeders in the choice experiment, they stayed longer on the control feeders than on the feeders with Roundup Ultra and this difference increased with concentration, here expressed in mg glyphosate/L (mg a.s./L). The predicted length of a visit to a Roundup feeder with 126 mg a.s./L was estimated to be 19 seconds, whereas a visit to a control feeder in the same trial was estimated to 66 seconds. Each circle shows the average length of a visit per trial and bumblebee individual. The position of the circles is jittered along the x axis to increase their visibility.

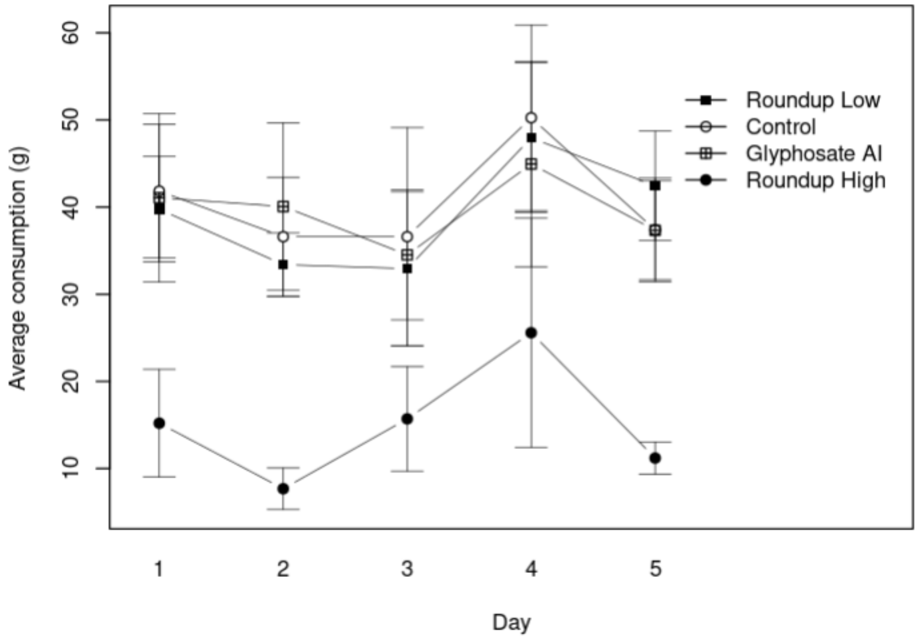

Figure S6 The average weight of sucrose solution consumed (g) from treated feeders over the 5-day period for colonies in the no choice experiment. Colonies consumed significantly less feed from the Roundup high treated feeders compared to all other treatments ($F_{3,15}= 1191, p= 0.0003$), but there was no significant interaction of treatment with ($F_{12,71} =0.16, p = 0.99$) or effect of day ($F_{4,83}=1.77, p=0.14$).
